# Association of Gabapentinoids With Opioid-Related Overdose in the Inpatient Setting: A Single Center Retrospective Case-Control Study

**DOI:** 10.1177/00185787231206522

**Published:** 2023-10-24

**Authors:** Shelby R. Humpert, Kelly R. Reveles, Kajal Bhakta, Sorina B. Torrez, Kirk E. Evoy

**Affiliations:** 1 The University of Texas at Austin, Austin, TX, USA; 2 University of Texas Health San Antonio, San Antonio, TX, USA; 3 University Health, San Antonio, TX, USA

**Keywords:** drug interactions, medication safety, pain management

## Abstract

**Objectives:** Recent data suggest concomitant gabapentinoid use increases opioid-related overdose (ORO) risk; however, this association has not been well studied in the hospital setting. The primary objective of this study was to compare ORO risk, indicated by naloxone administration, in patients receiving opioids plus gabapentinoids versus opioids alone. **Methods:** In this retrospective case-control study of adults admitted to a large community hospital from 1/1/20 to 12/31/21, all cases (defined as patients who received naloxone more than 24 hours after admission) identified were matched 1:1 to randomly selected controls (defined as patients on opioids who did not receive naloxone). The primary outcome was the percentage of cases and controls with concomitant inpatient gabapentinoid use. Logistic regression was performed to determine the independent association between gabapentinoids and ORO (as evidenced by inpatient naloxone administration). **Results:** Baseline characteristics were similar between the 144 cases and 144 controls. Gabapentinoid exposure was greater for cases than controls (34.0%vs 20.8%, *P* = .0118). Median hospital length of stay (11vs 4 days, *P* < .0001) and mortality (19%vs 5%; *P* = .0018) were also higher for cases. In logistic regression analysis, ORO (adjusted OR 4.91; 95% CI 1.86-12.96) and serotonergic medication exposure (adjusted OR 4.31; 95% CI 1.50-12.38) were significantly associated with gabapentinoid use. **Conclusions:** Concomitant gabapentinoid use with opioids was associated with increased ORO risk in the inpatient setting. When considering prescribing gabapentinoids in conjunction with opioids in the hospital setting, potential benefits should be weighed against increased overdose risk.

## Introduction

Annual opioid-related overdose (ORO) deaths continue to rise in the United States (US), including nearly 69 000 ORO deaths in 2020 alone, a 38% increase from 2019.^1^ Increased ORO risk with concomitant central nervous system (CNS) depressants, such as alcohol or benzodiazepines, is well-established.^2^ Gabapentin and pregabalin, referred to as gabapentinoids, collectively, are believed to downregulate glutamate release and antagonize N-methyl-D-aspartate (NMDA) receptors, blocking excitatory neurotransmission, leading to CNS depression.^3^ While originally approved by the US Food and Drug Administration (FDA) for indications such as seizure disorders and postherpetic neuralgia, these medications are commonly used off-label for many indications, including neuropathic pain, anxiety, and alcohol use disorder.^4,5^ In the hospital setting gabapentinoids are increasingly being used for pain management, especially post-surgery to reduce opioid consumption and increase patient satisfaction.^6-8^ Gabapentinoids are generally perceived as relatively low-risk medications for most patients and are typically well-tolerated.^9,10^ Single-substance gabapentinoid overdoses often cause sedation and nausea, but are rarely fatal.^3,11,12^ However, gabapentinoids potentiate the effects of opioids due to overlapping mechanisms that decrease excitatory neurotransmission and lead to CNS depression.^3^ Another suggested mechanism for potentiation is slowed gastric motility by opioids, which may increase absorption of gabapentinoids with longer gastrointestinal transit time.^13^ This potentiation may enhance pleasurable effects of opioids but also increases the risk of respiratory depression and fatal overdose.

A growing number of studies have shown increased patient harm associated with concomitant gabapentinoid and opioid use, including increased hospital/emergency services utilization, opioid overdose risk, and mortality.^13-16^ In 2019, the FDA released a statement warning that gabapentinoids may be linked to serious breathing difficulties, especially when used with opioids and other CNS depressants that cause respiratory depression, in people with reduced lung function, and in the elderly.^17^ Neither gabapentinoid has contraindications for respiratory concerns; however, respiratory depression is listed as a warning in the gabapentin prescribing information when used in combination with CNS depressants or with respiratory impairment.^9,10^

To date, most data establishing increased ORO risk with gabapentinoids were derived from large, retrospective, population-based studies.^14,15,18,19^ These often rely on prescription claims-based data or other healthcare or adverse event databases, where the precise overdose circumstances, such as use of concomitant drugs at the time of overdose, was unknown.^1-4^ There is limited evidence regarding the risks associated with this combination in the inpatient hospital setting in the US. The primary objective of this study was to assess the association of gabapentinoid use with ORO in the inpatient setting. It was hypothesized that concomitant gabapentinoid use would be more common among inpatients administered naloxone, the opioid reversal agent, compared to a control group.

## Methods

### Study Design

**Figure 1.**
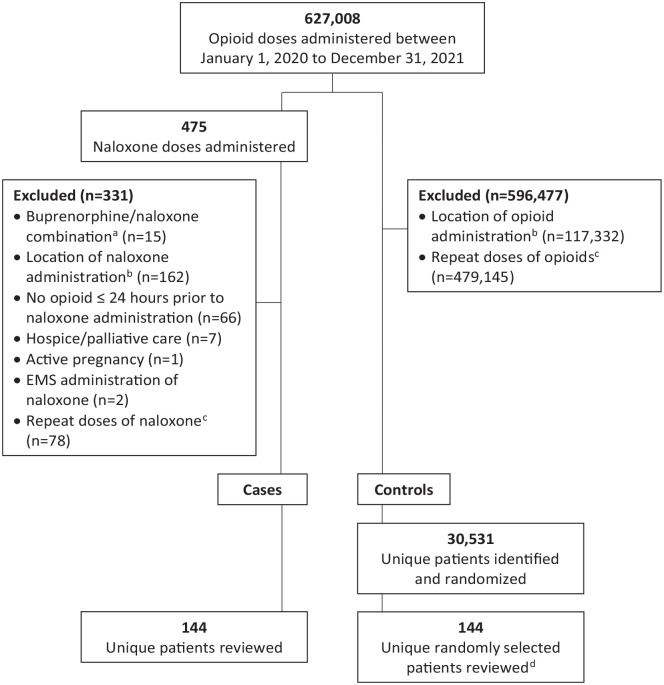
Selection of cases and controls. *Note.* EMS = emergency medical services. ^a^Buprenorphine/naloxone combination is indicated for the treatment of opioid use disorder, not used for opioid overdose reversal. ^b^Locations where naloxone/opioid were administered that were removed to apply exclusion criteria include: emergency department, surgical admissions (outpatient visits), bone marrow transplant (outpatient visits), rehabilitation (not considered admitted), labor and delivery, antepartum, mother and baby care. ^c^Initial administration of naloxone/opioid selected as index administration to identify unique patients and administrations at subsequent hospital admissions removed to prevent duplication of cases/controls. ^d^The first 144 randomized controls that met inclusion criteria were selected to provide an equivalent number of controls to case patients.

This was a single-center, retrospective, case-control study of inpatients admitted to an 800-bed community hospital in San Antonio, Texas from January 1, 2020 to December 31, 2021. Patients were eligible for inclusion if they were at least 18 years old, had documented administration of opioids (buprenorphine, codeine, fentanyl, hydrocodone, hydromorphone, meperidine, methadone, morphine, oxycodone, oxymorphone, or tramadol; oral, intravenous, and transdermal patch routes of administration) during current hospitalization, and at least 24 hours of opioid use. Patients were excluded if they were pregnant or if they received naloxone and/or an opioid in the emergency department only (ie, did not receive naloxone or an opioid while admitted to the hospital). Patients receiving palliative care/hospice services were also excluded to minimize inclusion of patients receiving elevated doses of opioids for comfort care, and in which the naloxone administration may be avoided. All cases, defined as those who received at least one dose of naloxone during the study period, were included, as well as an equivalent number of control patients, defined as those who received an opioid but did not receive naloxone (control group) any time during admission. Naloxone administration was used as a surrogate marker for ORO for the cases, as suspected opioid overdose would be the only indication for medication administration. The naloxone group sample size was based on access and feasibility and an equivalent size control group was used. An a priori sample size calculation that used an estimated 15% effect size, alpha of 0.05, and power of 80% gave an estimated sample size of 120 people per group needed. Figure 1 illustrates the applied inclusion/exclusion criteria to cases and controls. Initial lists were gathered from medication administration software, filtered for exclusion criteria, and converted from medication administrations to individual patients by identifying and condensing multiple administrations for the same patient. The control patients were randomized and if one was found to not meet criteria, the next patient on the list was reviewed. The hospital’s Institutional Review Board granted approval for this study (protocol number: 1877878). Protected health information (PHI) was deidentified in the data collection tool and password-protected to ensure adequate privacy protection.

### Data Collection

Patient data were collected via manual chart review from the Meditech^®^ (Medical Information Technology Incorporated, Westwood, MA, USA) electronic health record (EHR) and recorded in Microsoft Excel^®^ (Microsoft Corporation, Redmond, WA, USA). Administration of naloxone and opioids was identified using VigiLanz^®^ (VigiLanz Corporation, Minneapolis, MN, USA) surveillance software to obtain initial patient lists.

Demographic information such as age, sex, race/ethnicity, height, and weight were recorded along with hospital length of stay (LOS), surgery within 24 hours of naloxone/opioid administration, and inpatient mortality status. The most recent lab values prior to naloxone or opioid administration were recorded to assess kidney (estimated glomerular filtration rate [eGFR], serum creatinine) and liver function (aspartate aminotransferase [AST], alanine aminotransferase [ALT], total protein, albumin, and bilirubin). If lab values were not available prior to naloxone or opioid administration, the soonest values after administration were recorded. Smoking status and the documentation of select comorbid conditions that may impact patient respiratory status, overdose risk, or medication kinetics (opioid use disorder, substance use disorder, active COVID-19 infection, hepatitis, heart failure, chronic obstructive pulmonary disease [COPD], obstructive sleep apnea [OSA], asthma, pneumonia) were obtained from admission intake, history and physical, and provider progress notes, as available. Diagnoses that were not documented in the chart were assumed to have not occurred. Home opioid use prior to admission was obtained from medication reconciliation lists or progress notes, as available.

The primary outcome of gabapentinoid exposure was defined as administration of gabapentin or pregabalin within the 24 hours preceding naloxone administration for cases and 24 hours after any opioid administration during the hospitalization for controls. This timeframe was chosen in an attempt to determine if an association exists when opioids and gabapentinoids are given concomitantly and based on pharmacokinetic parameters (eg, time-to-peak concentration). During the timeframe, drug name, dose, and number of administrations were recorded for gabapentinoids, opioids, and other sedating medications that could potentially contribute to overdose risk including benzodiazepines, Z-hypnotic sleep medications, muscle relaxants, first generation antihistamines, antipsychotics, anticonvulsants, and serotonergic agents. Serotonergic agents included receipt of at least one of the following: selective serotonin reuptake inhibitors (SSRI), serotonin-norepinephrine reuptake inhibitors (SNRI), serotonin modulators (eg, trazodone), and tricyclic antidepressants (TCA). While indications for use may overlap among the drug classes under investigation, benzodiazepines were specifically grouped separately due to the known increased risk of opioid overdose when benzodiazepines are used concomitantly with opioids.^8^ Morphine milliequivalents (MME) for the 24-hour window were calculated using Excel. For those using patient-controlled analgesia (PCA) pumps or drips, the maximum amount of opioid that the patient could receive was used, as there was no way to determine the exact quantity of medication received from the EHR due to the “as needed” nature of those medications. Median opioid doses during the 24-hour window were calculated, and these median doses were also stratified into categories (eg, MME <50, ≥50-90, ≥90-200, ≥200). These categories were selected based on clinical guidelines and previous research showing dose-dependent increased risk of ORO.^8^ Similarly, gabapentinoids were stratified into high (gabapentin ≥ 1800 mg/24 hours; pregabalin ≥ 300 mg/24 hours) or low (gabapentin < 1800 mg/24 hours; pregabalin < 300 mg/24 hours) dose categories based on common dosing ranges and prior research.^9,10,14,15^ Prior SUD and OUD diagnoses were defined through documentation in provider notes.

### Data Analysis

Data analysis was conducted using Excel and JMP Pro 17^®^ (SAS Institute, Cary, NC, USA). Baseline characteristics were compared between cases and controls using descriptive statistics, as well as chi-square, Wilcoxon rank sum, and Fisher’s exact test, as appropriate. Logistic regression was performed to determine the independent association between concomitant gabapentinoids and ORO. First, unadjusted odds ratios (OR) were calculated to determine the relationship of baseline characteristics to concomitant gabapentinoids. Characteristics with a *P*-value of < .2 in univariate analyses were selected as covariates for logistic regression and adjusted OR were reported. A *P*-value of <.05 was considered statistically significant for final analyses. Missing data were excluded from analyses.

## Results

In total, 288 unique patients were included: 144 cases who met inclusion criteria and received naloxone (naloxone group) and 144 controls (no naloxone group). Baseline characteristics were similar between groups for most characteristics, with notable differences in median age, location in hospital during medication administration, prior surgery, renal and liver function, and concomitant use of first generation antihistamines and antipsychotics (Table 1). While median age was slightly higher for the cases (66vs 61 years, *P* = .0086), there was no difference between groups in the proportion of patients ≥65 years old. Several liver function labs were different between groups, however all were within the normal ranges.

**Table 1.** Baseline Characteristics.

Characteristic	Cases (n = 144)	Controls (n = 144)	*P*-value
Age, median (IQR)	66 (55-75)	61 (44-71)	.**0086**
Age ≥65 y, n (%)	76 (52.8)	60 (41.7)	.0587
Female sex, n (%)	79 (54.9)	88 (61.1)	.2824
Race, n (%)			.3390
Not Hispanic or Latino	83 (57.6)	73 (50.7)	
Hispanic or Latino	51 (35.4)	57 (39.6)	
Not documented	10 (6.9)	14 (9.7)	
BMI (kg/m^2^), median (IQR)	27.9 (24.2-33.1)	29.1 (24.1-33.8)	.4849
Obesity category,^a^ n (%)			.4054
Underweight	6 (4.2)	2 (1.4)	
Normal	34 (23.6)	36 (25.0)	
Overweight	46 (31.9)	40 (27.8)	
Obese	58 (40.2)	66 (45.8)	
Location, n (%)			.**0003**
Medicine	88 (61.1)	116 (80.6)	
ICU	56 (38.9)	28 (19.4)	
Prior surgery,^b^ n (%)	38 (27.3)	62 (44.6)	.**0026**
Smoking status, n (%)			.4455
Current	13 (9.0)	13 (9.0)	
Former	27 (18.8)	18 (12.5)	
Denied	74 (51.3)	76 (52.8)	
Not documented	30 (20.8)	14 (9.7)	
Opioids prior to admission, n (%)	33 (22.9)	34 (23.6)	.5603
MME (mg/day), median (IQR)	30 (15-82)	32.3 (15.3-62.6)	.8846
MME category (mg/day), n (%)			.0817
<50 MME	90 (62.5)	98 (68.1)	
50 − <90 MME	21 (14.6)	26 (18.1)	
90 − <199 MME	21 (14.6)	15 (10.4)	
≥200 MME	12 (8.3)	5 (3.5)	
Other sedating medications, n (%)			
Any sedating medication	62 (43.1)	47 (32.6)	.0681
Serotonergic agents^c^	19 (13.2)	15 (10.4)	.4646
Benzodiazepines	27 (18.8)	27 (18.8)	1.0000
Z-hypnotics	1 (0.7)	1 (0.7)	1.0000
Muscle relaxants	1 (0.7)	3 (2.1)	.3031
First generation antihistamines	16 (11.1)	7 (4.9)	.**0476**
Antipsychotics	19 (13.2)	1 (0.7)	<.**0001**
Anticonvulsants	4 (2.8)	0 (0)	.**0179**
SUD, n (%)	14 (12.6)	11 (12.6)	.5237
OUD, n (%)	4 (3.6)	4 (3.6)	1.0000
Renal function			
GFR ≥60^d^, n (%)	66 (47.1)	82 (72.6)	<.**0001**
CrCl,^e^ median (IQR)	52.7 (26.5-83.1)	69.4 (47.7-112.6)	<.**0001**
Liver function labs, median (IQR)			
AST (units/L)	32 (16-57)	21 (16-39.8)	.**0437**
ALT (units/L)	27 (16-48)	28 (18-46)	.5341
Total protein (g/dL)	6.5 (5.7-7.1)	6.9 (6.1-7.4)	.**0084**
Albumin (g/dL)	2.6 (2.2-3.1)	3.1 (2.6-3.5)	<.**0001**
Total bilirubin (mg/dL)	0.6 (0.4-1.0)	0.6 (0.4-0.8)	.2820
Select comorbid conditions, n (%)			
COVID-19 positive	6 (5.3)	2 (2.0)	.2883
Hepatitis	2 (1.4)	3 (2.1)	1.0000
Heart failure	21 (14.6)	16 (11.1)	.3780
COPD	13 (9.0)	11 (7.6)	.6697
OSA	5 (3.5)	5 (3.5)	1.0000
Asthma	5 (3.5)	3 (2.1)	.7227
Pneumonia	17 (11.8)	4 (2.8)	.**0053**

*Note.* BMI = body mass index; ICU = intensive care unit; MME = morphine milligram equivalents; SUD = substance use disorder; OUD = opioid use disorder; GFR = glomerular filtration rate; CrCl = creatinine clearance; AST = aspartate aminotransferase; ALT = alanine aminotransaminase; COPD = chronic obstructive pulmonary disease; OSA = obstructive sleep apnea. Bold text indicates statistical significance.

a Underweight = BMI < 18.5 kg/m^2^; Normal weight = BMI 18.5-24.9 kg/m^2^; Overweight = BMI 25.0-29.9 kg/m^2^; Obese = BMI ≥ 30 kg/m^2^.

b Defined as surgical procedure 24 hours prior to naloxone administration (cases) or in the 24-hour opioid window (controls).

c Selective serotonin reuptake inhibitors (SSRI), serotonin norepinephrine reuptake inhibitors (SNRI), serotonin modulators, tricyclic antidepressants (TCA).

d Any GFR ≥ 60 mL/min/1.73m^2^ was reported as “≥60”.

e Calculated using Cockcroft-Gault formula in mL/min.

The primary outcome of gabapentinoid exposure was significantly greater among cases with 49 patients (34.0%) having received a gabapentinoid ≤ 24 hours prior to naloxone administration versus 30 patients (20.8%, *P* = .0118) having received a gabapentinoid within 24 hours of any opioid administration in the control group. No significant difference was observed in high or low dosing of gabapentinoids between the cases and the controls, though there were few patients receiving high doses with only 3 (6.1%) and 1 (3.3%; *P* = .5723) receiving high dose in the case and control groups, respectively. Median hospital LOS (11vs 4 days; *P* < .0001) and inpatient mortality (19%vs 5%; *P* = .0018) were higher for cases (Table 2).

**Table 2.** Outcomes by Group.

Outcome	Cases (n = 144)	Controls (n = 144)	*P*-value
Opioid only, n (%)	95 (66.0)	114 (79.2)	.**0118**
Gabapentinoid plus opioid, n (%)	49 (34.0)	30 (20.8)	
Gabapentin, n (%)	43 (29.9)	27 (18.8)	.**0274**
Pregabalin, n (%)	6 (4.2)	3 (2.1)	.5008
Gabapentinoid dose,^a^ median (IQR)			
Gabapentin	600 (300-600)	600 (300-800)	.6123
Pregabalin	62.5 (50-225)	100 (75-100)	.5078
Gabapentin dose, n (%)			.5723
High (≥1800 mg)	2 (4.7)	1 (3.7)	
Low (<1800 mg)	41 (95.3)	26 (96.3)	
Pregabalin dose, n (%)			.1389
High (≥300 mg)	1 (16.7)	0 (0)	
Low/Moderate (<300 mg)	5 (83.3)	3 (100)	
High dose of either gabapentinoid, n (%)	3 (6.1)	1 (3.3)	.5723
Hospital LOS, median (IQR)	11 (7-23)	4 (2-8)	<.**0001**
Mortality, n (%)	19 (14.1)	5 (3.7)	.**0018**

*Note.* LOS = length of stay. Bold text indicates statistical significance.

a Milligrams of gabapentinoid administered within 24 hours prior to naloxone administration (cases) or within 24 hours after opioid administration (controls).

Independent predictors of gabapentinoid exposure can be seen in Table 3. Naloxone (case group), female sex, total protein and bilirubin labs, history of SUD, and concomitant use of serotonergic agents, antipsychotics, and anticonvulsants were characteristics included in the multivariable model based on unadjusted odds ratios and univariate *P*-value cutoff. In the multivariable model, naloxone was a significant predictor of gabapentinoid use (adjusted OR 4.91; 95% CI 1.86-12.96), as was concomitant serotonergic agents (adjusted OR 4.31; 95% CI 1.50-12.38) and anticonvulsants (adjusted OR 21.87; 95% CI 1.06-449.78).

**Table 3.** Independent Predictors of Concomitant Gabapentinoid Use.

Characteristic	Unadjusted OR (95% CI)	*P*-value	Adjusted OR (95% CI)	*P*-value
Naloxone (case group)	1.96 (1.15-3.33)	.0128*	4.91 (1.86-12.96)	.**0013**
Age	1.00 (0.99-1.03)	.2526		
Age ≥65 y	0.98 (0.58-1.64)	.9356		
Female sex	1.46 (0.85-2.50)	.1624*	1.00 (0.44-2.26)	.9949
Race				
Not Hispanic or Latino	1.0 (reference)			
Hispanic or Latino	0.96 (0.56-1.65)	.8905		
BMI	1.00 (0.97-1.04)	.7823		
Overweight	0.95 (0.53-1.69)	.8575		
Location				
Medicine	1.0 (reference)			
ICU	0.77 (0.43-1.38)	.3775		
Prior surgery^a^	1.21 (0.70-2.09)	.5010		
Smoking ever	0.83 (0.43-1.63)	.5950		
Opioids prior to admission	1.31 (0.68-2.54)	.4226		
MME	1.00 (1.00-1.00)	.9068		
MME category				
<50 MME	1.0 (reference)			
50-<90 MME	1.41 (0.70-2.82)	.3374		
90-<199 MME	1.50 (0.70-3.23)	.3005		
≥200 MME	1.25 (0.42-3.73)	.6894		
Other sedating medications				
Any sedating medication	1.56 (0.92-2.64)	.0978		
Serotonergic agents^b^	2.69 (1.30-5.60)	.0079*	4.31 (1.50-12.38)	.**0066**
Benzodiazepines	1.14 (0.60-2.19)	.6880		
Z-hypnotics	6.54e-7 (0)	.9920		
Muscle relaxants	0.88 (0.09-8.60)	.9127		
Antihistamines	0.93 (0.35-2.45)	.8803		
Antipsychotics	2.31 (0.92-5.82)	.0745*	1.10 (0.24-5.04)	.9024
Anticonvulsants	8.21 (0.84-80.14)	.0701*	21.87 (1.06-449.78)	.**0455**
SUD	0.39 (0.11-1.36)	.1395*	0.12 (0.01-1.16)	.0559
OUD	2.71e-7 (0)	.9903		
Renal function				
GFR ≥60^c^	0.92 (0.52-1.62)	.7650		
CrCl^d^	1.00 (0.99-1.01)	.7972		
Liver function				
AST	1.00 (1.00-1.00)	.9682		
ALT	1.00 (1.00-1.00)	.7073		
Total protein	0.75 (0.57-0.99)	.0415*		
Albumin^e^	0.67 (0.44-1.01)	.0510		
Total bilirubin	0.81 (0.58-1.11)	.1096*		
Comorbid conditions				
COVID-19 positive	0.89 (0.18-4.55)	.8916		
Hepatitis	2.37e-7 (0)	.9918		
Heart failure	1.14 (0.53-2.43)	.7372		
COPD	0.87 (0.33-2.28)	.7806		
OSA	0.65 (0.14-3.14)	.5945		
Asthma	1.61 (0.38-6.90)	.5210		
Pneumonia	0.82 (0.29-2.30)	.6997		

*Note.* BMI = body mass index; ICU = intensive care unit; MME = morphine milligram equivalents; SUD = substance use disorder; OUD = opioid use disorder; GFR = glomerular filtration rate; CrCl = creatinine clearance; AST = aspartate aminotransferase; ALT = alanine aminotransaminase; COPD = chronic obstructive pulmonary disease; OSA = obstructive sleep apnea. Bold text indicates statistical significance.

a Defined as surgical procedure 24 hours prior to naloxone administration (cases) or in the 24-hour opioid window (controls).

b Selective serotonin reuptake inhibitors (SSRI), serotonin norepinephrine reuptake inhibitors (SNRI), serotonin modulators, tricyclic antidepressants (TCA).

c Any GFR ≥ 60 mL/min/1.73m^2^ was reported as “≥60”.

d Calculated using Cockcroft-Gault formula in mL/min.

e Albumin not included in regression model as it is a component of total protein measurement.

* Characteristic selected as covariate included in logistic regression model with *P*-value of <.2.

## Discussion

This study identified a significant association between gabapentinoid exposure and ORO, indicated by naloxone administration, in adults admitted to the hospital. The odds of gabapentinoid exposure were 4.9 times higher in patients who received naloxone for an ORO compared to those who did not. Hospital LOS and mortality were also higher for cases. This represents one of the first studies to identify higher concomitant use of gabapentinoids with opioids in patients who experience ORO in the inpatient hospital setting.

This study contributes to the growing body of evidence regarding potential harms associated with concomitant use of gabapentinoids with opioids identified in previous studies.^12,14,15^ Much of the current evidence regarding ORO risk and other harms associated with concomitant gabapentinoid use is based on insurance, medical record, prescription, and post-mortem toxicology databases. Gomes et al^14,15^ identified increased mortality risk in patients prescribed both an opioid and gabapentinoid within 120 days prior to death versus those receiving opioids alone. This risk was 1.49 times higher with gabapentin and 1.68 times higher with pregabalin, and the relationship was dose-dependent, with greater risk observed in patients on higher gabapentinoid doses. Khan et al^16^ also found gabapentin associated with a 1.16 times higher risk of opioid overdose utilizing insurance claims data. Mortality risk with gabapentinoids has also been studied in patients receiving opioid agonist therapy (OAT; eg, buprenorphine or methadone) for opioid use disorder, using mortality and prescription databases.^18,19^ Macleod et al found that patients co-prescribed gabapentinoids during OAT, and up to 12 months post-treatment, were 1.71 times more likely to experience death from any cause compared to those without gabapentinoid exposure. Similarly, Abrahamsson et al found that overlapping daily doses of OAT and pregabalin were associated with 2.82 times increased risk of ORO death. While these studies are important in illustrating the harms associated with concurrent gabapentinoid and opioid use in the outpatient setting, these population-level data have limitations regarding the precise medications, prescription or other, and doses the patient took prior to overdose/death, as well as patient characteristics and health status. Furthermore, this risk in the outpatient setting may not be reflective of the risk when gabapentinoids and opioids are administered concomitantly during hospitalization.

Few studies have been conducted to assess the risks associated with concomitant gabapentinoid and opioid use in the inpatient setting. Two studies analyzed characteristics of patients receiving opioids with gabapentinoids in the inpatient setting, but neither included a control group of patients not receiving naloxone.^20,21^ Because all of these patients received naloxone for a suspected overdose, there are not really any meaningful conclusions that can be drawn from these studies with regards to relative ORO risk associated with gabapentinoids. It is also worth noting that these studies were small and may have been underpowered to detect differences in respiratory depression/sedation with gabapentinoid exposure. Another study assessed safety outcomes of gabapentinoid use with or without opioid exposure and found no differences in sedation scores or adverse event rates of ataxia, confusion, dizziness, or blurred vision.^22^ This study did not assess ORO or naloxone administration, so, similarly, no conclusions about relative ORO risk can be drawn.

The best current evidence for gabapentinoids increasing ORO risk in the inpatient setting was documented by Minhaj et al^23^ Similar to the present study, naloxone was utilized as a surrogate marker for opioid overdose and risk factors for opioid-related adverse drug events (ORADE) requiring naloxone were assessed in inpatients from a single US hospital, finding gabapentinoids were associated with a 1.67 times increased risk of ORADE. While this retrospective case-control study had a naloxone and a no naloxone group, unlike the current study, gabapentinoid exposure prior to naloxone administration was not the primary outcome under investigation, timing of gabapentinoid administration in relation to naloxone administration was not addressed in the manuscript, and gabapentinoid and opioid doses were not compared between groups. The present study corroborates these findings from Minhaj et al and builds upon previous research identifying an association between increased ORO risk and concomitant gabapentinoid and opioid use.^13-16,18,19,23^ This study was designed specifically to assess associations related to overdose in the inpatient setting. Patients who received naloxone in the emergency department were excluded, as this would more likely be the result of an outpatient overdose prior to admission.

In addition to assessing recent gabapentinoid exposure, this study also evaluated opioid and gabapentinoid dosing, as well as recent exposure to other potentially sedating medications to address potential confounders. Gabapentinoid dosing is of interest given higher doses have been shown to further increase risk of adverse effects and overdose risk, especially when given with opioids or other CNS depressants and in older adults with comorbidities such as chronic kidney disease .^2,24,25^ The aforementioned Gomes et al studies identified a gabapentinoid-dose-dependent risk.^14,15^ Among people prescribed opioids who died from an opioid-related cause, moderate (900-1799 mg daily) and high (≥1800 mg daily) doses of gabapentin were associated with a nearly 60% increase in the odds of opioid-related death compared to groups with no concomitant gabapentin use. Other studies have shown increased risk of hospital utilization, ORO, and mortality with higher doses of gabapentinoids, particularly with higher doses of opioids (>50 MME).^13-15^ In the present study, no significant difference in median gabapentinoid doses between the naloxone and control groups was observed, though there were so few patients receiving high-dose gabapentinoids, this study was likely not powered to detect a difference. The lack of high doses could be due to several factors, including the fact that gabapentinoids are typically given at low doses initially and titrated up as needed over time. Unless the patient was on relatively high dose gabapentinoids prior to hospitalization, the doses initiated in the hospital would likely be relatively low. Also, lower doses are recommended in patients with poor renal function as gabapentinoids are renally eliminated.^9,10^ The cases in this study had worse renal function compared to the control group (creatinine clearance 53 mL/min vs 69 mL/min; *P* < .0001), however the median gabapentin dose of 600 mg per day seen in this study can be used in patients with creatinine clearance as low at 15 mL/min.^9^ Additional research is needed to assess if is there is a correlation between gabapentinoid dose and ORO risk in the hospital setting.

Opioid doses were also not significantly different between groups. The median MME was about 30 mg daily for both groups and no significant differences were observed when opioid doses were stratified (eg, MME <50, ≥50-90, ≥90-200, ≥200), so it does not appear that differences between cases and controls resulted from differences in opioid utilization.^8,13^ Recent exposure to other potentially sedating medications was also recorded, as all opioids carry a warning for risk of sedation, respiratory depression, coma, and death when concomitantly used with benzodiazepines or other CNS depressants.^2^ No between-group difference was observed in recent exposure to sedating medications collectively; however, there were significantly more patients who received first-generation antihistamines or antipsychotics in the naloxone group. Interestingly, Minhaj et al ^23^ found a negative association between antihistamines and naloxone use. Besides gabapentinoids, Minhaj et al did not identify any additional medications associated with an increased likelihood of naloxone administration, including benzodiazepines, muscle relaxants, or tricyclic antidepressants. In the present study, serotonergic agents and anticonvulsants were significant covariates associated with concomitant gabapentinoid use. Serotonergic agents are potentially related to gabapentinoid use due to overlapping indications, such as neuropathic pain management between the gabapentinoids and the SNRIs, or possibly off-label indication of gabapentinoids for anxiety.^26^ While anticonvulsant use was associated, only 4 patients received an anticonvulsant and the point estimate is likely imprecise, evidenced by the large confidence interval (adjusted OR 21.87; 95% CI 1.06-449.78), so further research is needed to better assess this possible association.

Hospital LOS and mortality were significantly higher in the naloxone group, corroborating naloxone administration as an important surrogate marker for ORO and subsequent negative consequences, such as death or need for extended care. It is difficult to determine the cause of the increased length of stay and higher mortality in the naloxone group from the information available, but it could be related to opioid-overdose effects, higher acuity of illness, as evidenced by more patients in that naloxone group in the ICU, or due to other factors the present study was not designed to investigate. Minhaj et al^23^ similarly found that naloxone administration was associated with increased LOS. Of note, that study excluded patients in the ICU, a group that made up a large proportion of the present study’s population. However, the present study did not assess the movement of patients to or from the ICU or medical units, so the relative time patients spent in one unit or another during the course of their admission could not be elucidated. Because the naloxone group included both patients who did and did not receive gabapentinoids, it is not possible to extrapolate LOS and mortality findings to gabapentinoid use specifically. However, it is important to recognize the consequences of ORO in hospitalized patients, especially in more critically ill patients, and to take preventative measures to help prevent these events, which may include cautiously prescribing the combination of opioids with gabapentinoids.

### Limitations

There are several potential limitations of this study. First, a case-control design was necessary given the uncommon outcome of inpatient naloxone administration. Case-control designs are subject to several types of bias and are unable to determine outcome incidence. Data were obtained retrospectively from the hospital EHR, and the data quality is dependent on what was documented in the chart during the patient’s admission. While medication administration information was well documented due to barcoding technology, other things, like history and physical notes, home medication lists, and progress notes were not standardized across records. Patients with a prior history of SUD/OUD or buprenorphine/methadone were not excluded, so as to optimize the sample size, though these patients may have biased to a larger naloxone administration event rate. Given the similarity between case and control cohorts though, the impact of these diagnoses and medications on the overall findings is expected to be minimal. The calculated MME may represent an overestimation of actual amount of opioid received for patients receiving PCA, since the precise amount of opioid received from a PCA or drip was not known, and therefore the maximum amount allowed was recorded. However, the average MME was similar between groups. Race and ethnicity categories were not consistently documented and the classification of “Hispanic or Latino” or “not Hispanic or Latino” were the most commonly reported descriptions. While baseline characteristics were largely similar, there was a statistically significant difference between those receiving naloxone and those not receiving naloxone in several key factors, including age, ICU status, and kidney function, that may have impacted the results. Another limitation was that few patients received high-dose gabapentinoids or opioids, so the relationship between dose and ORO risk cannot be accurately assessed in this study. Hospital LOS and mortality outcomes may be highly influenced by confounding factors that may also play a role in ORO so these findings should be viewed as exploratory. Due to small sample size, point estimates for some characteristics in the regression model may be imprecise, as demonstrated with large confidence intervals. Finally, naloxone was used as a surrogate marker for opioid overdose. Due to documentation limitations, it was not possible to confirm overdose with other indicators such as respiratory depression, Pasero opioid-induced sedation (POSS) scale, or opioid overdose diagnosis codes. Naloxone administration has been used in many other studies as an indicator of opioid overdose, but its use alone may be an underestimate of overdose events.^27^

## Conclusions

In this single-center, retrospective, case-control study, concomitant gabapentinoid use with opioids was associated with increased ORO risk in the inpatient hospital setting. When considering prescribing gabapentinoids in conjunction with opioids in the hospital, potential benefits should be weighed against increased overdose risk. Larger, prospective, more geographically diverse studies are warranted to confirm these findings and better inform practitioners and protect patient safety.

## References

[1] Centers for Disease Control and Prevention. Understanding the Opioid Overdose Epidemic. Centers for Disease Control and Prevention. Updated June 1, 2022. Accessed February 1, 2023. https://www.cdc.gov/opioids/basics/epidemic.html

[bibr2-00185787231206522] US Food and Drug Administration. FDA Requires Strong Warnings for Opioid Analgesics, Prescription Opioid Cough Products, and Benzodiazepine Labeling Related to Serious Risks and Death From Combined Use. News Release. US Food and Drug Administration. August 31, 2016. Accessed January 15, 2023. https://www.fda.gov/news-events/press-announcements/fda-requires-strong-warnings-opioid-analgesics-prescription-opioid-cough-products-and-benzodiazepine 10.1080/15360288.2016.1241336 27960626

[bibr3-00185787231206522] Evoy KE Peckham AM Covvey JR Tidgewell KJ. Gabapentinoid pharmacology in the context of emerging misuse liability. J Clin Pharmacol. 2021;61(S2):S89-S99. doi:10.1002/jcph.1833 34396549

[4] Gabapentin. Lexi-Drugs. Lexicomp, 2022. Updated March 9, 2023.

[5] Pregabalin. Lexi-Drugs. Lexicomp, 2015. Updated March 2, 2023.

[6] Ladich EM Zhou KQ Spence DL Moore CB. Opioid-sparing anesthesia: gabapentin and postoperative pain. J Perianesth Nurs. 2022;37(6):966-970. doi:10.1016/j.jopan.2022.04.008 36100525

[bibr7-00185787231206522] Wick EC Grant MC Wu CL. Postoperative multimodal analgesia pain management with nonopioid analgesics and techniques a review. JAMA Surg. 2017;152(7):691-697. doi:10.1001/jamasurg.2017.0898 28564673

[8] Dowell D Haegerich TM Chou R. CDC guideline for prescribing opioids for chronic pain-United States, 2016. JAMA. 2016;315(15):1624-1645. doi:10.1001/jama.2016.1464 26977696 PMC6390846

[9] Gabapentin [package insert]. Pfizer Inc; 1993.

[10] Pregabalin [package insert]. Pfizer Inc; 2004.

[11] Evoy KE Morrison MD Saklad SR. Abuse and misuse of pregabalin and gabapentin. Drugs. 2017;77(4):403-426. doi:10.1007/s40265-017-0700-x 28144823

[12] Evoy KE Sadrameli S Contreras J , et al. Abuse and misuse of pregabalin and gabapentin: a systematic review update. Drugs. 2021;81(1):125-156. doi:10.1007/s40265-020-01432-7 33215352

[13] Peckham AM Fairman KA Sclar DA. All-cause and drug-related medical events associated with overuse of gabapentin and/or opioid medications: a retrospective cohort analysis of a commercially insured US population. Drug Safety. 2018;41(2):213-228. doi:10.1007/s40264-017-0595-1 28956286

[bibr14-00185787231206522] Gomes T Juurlink DN Antoniou T , et al. Gabapentin, opioids, and the risk of opioid-related death: a population-based nested case–control study. PLoS Med. 2017;14(10):e1002396. 10.1371/journal.pmed.1002396 PMC5626029 28972983

[bibr15-00185787231206522] Gomes T Greaves S van den Brink W , et al. Pregabalin and the risk for opioid-related death: a nested case–control study. Ann Intern Med. 2018;169(10):732-734. doi:10.7326/m18-1136 30140853

[16] Khan NF Bykov K Glynn RJ Barnett ML Gagne JJ. Coprescription of opioids with other medications and risk of opioid overdose. Clin Pharmacol Ther. 2021;110(4):1011-1017. doi:10.1002/cpt.2314 34048030

[17] FDA warns about serious breathing problems with seizure and nerve pain medicines gabapentin (Neurontin, Gralise, Horizant) and pregabalin (Lyrica, Lyrica CR) U.S. Food and Drug Administration. Drug Safety Communication. December 19, 2019. Accessed February 1, 2023. https://www.fda.gov/drugs/drug-safety-and-availability/fda-warns-about-serious-breathing-problems-seizure-and-nerve-pain-medicines-gabapentin-Neurontin

[18] Macleod J Steer C Tilling K , et al. Prescription of benzodiazepines, z-drugs, and gabapentinoids and mortality risk in people receiving opioid agonist treatment: observational study based on the UK Clinical Practice Research Datalink and Office for National Statistics death records. PLoS Med. 2019;16(11):e1002965. doi:10.1371/journal.pmed.1002965 10.1371/journal.pmed.1002965 PMC6879111 31770388

[19] Abrahamsson T Berge J Öjehagen A Håkansson A. Benzodiazepine, z-drug and pregabalin prescriptions and mortality among patients in opioid maintenance treatment—a nation-wide register-based open cohort study. Drug Alcohol Depend. 2017;174:58-64. doi:10.1016/j.drugalcdep.2017.01.013 28315808

[20] Desai PH Taylor O Shah KJ Evoy KE Peckham AM. Characterization of hospitalized patients who received naloxone while receiving opioids with or without gabapentinoids. Ment Health Clin. 2021;11(4):225-230. doi:10.9740/mhc.2021.07.225 34316417 PMC8287869

[21] Savelloni J Gunter H Lee KC , et al. Risk of respiratory depression with opioids and concomitant gabapentinoids. J Pain Res. 2017;10:2635-3641. doi:10.2147/JPR.S144963 29180889 PMC5691933

[22] Tu TG Sadeghi S Atayee R Lee K. Gabapentinoid dosing and associated toxicities in patients with or without concomitant opioids during hospitalization. Am J Hosp Palliat Med. 2022;39(5):530-534. doi:10.1177/10499091211040231 34409886

[23] Minhaj FS Rappaport SH Foster J Gashlin LZ. Predictors of serious opioid-related adverse drug events in hospitalized patients. J Patient Saf. 2021;17(8):e1585-e1588. doi:10.1097/PTS.0000000000000735 32502115

[24] Muanda FT Weir MA Ahmadi F , et al. Higher-dose gabapentinoids and the risk of adverse events in older adults with CKD: a population-based cohort study. Am J Kidney Dis. 2022;80(1):98-107.e1. doi:10.1053/j.ajkd.2021.11.007 34979160

[25] Olopoenia A Camelo-Castillo W Qato DM , et al. Adverse outcomes associated with concurrent gabapentin, opioid, and benzodiazepine utilization: a nested case-control study. Lancet Reg Heal Am. 2022;13:100302. doi:10.1016/j.lana.2022.100302 PMC9904085 36777316

[26] Bates D Schultheis BC Hanes MC , et al. A comprehensive algorithm for management of neuropathic pain. Pain Med. 2019;20:S2-S12. doi:10.1093/pm/pnz075 PMC6544553 31152178

[27] Danovitch I Vanle B Van Groningen N Ishak W Nuckols T. Opioid overdose in the hospital setting: a systematic review. J Addict Med. 2020;14(1):39-47. doi:10.1097/ADM.0000000000000536 30950913

